# Sex and Gender Differences in Psychosocial Risk Profiles Among Patients with Coronary Heart Disease — the THORESCI-Gender Study

**DOI:** 10.1007/s12529-023-10170-5

**Published:** 2023-05-11

**Authors:** Sophie C. M. van den Houdt, Paula M. C. Mommersteeg, Jos Widdershoven, Nina Kupper

**Affiliations:** 1 Center of Research On Psychological Disorders and Somatic Diseases (CoRPS), Department of Medical & Clinical Psychology, Tilburg University, PO Box 90153, 5000 LE Tilburg, the Netherlands; 2 Department of Cardiology, Elisabeth-TweeSteden Hospital, Doctor Deelenlaan 5, 5042 AD Tilburg, the Netherlands

**Keywords:** Psychosocial risk factors, Gender, Sex, Acute coronary syndrome; Latent profile analysis

## Abstract

**Background:**

Psychosocial factors tend to cluster and exhibit differences associated with sex assigned at birth. Gender disparities, though, remain uncharted so far. The current study aimed to first explore the clustering of eight established psychosocial risk factors among patients with coronary heart disease (CHD), followed by examining how sex and gender differences characterize these psychosocial risk profiles, while adjusting for the effect of age.

**Method:**

In total, 532 patients with CHD (*M*_age_ = 68.2 ± 8.9; 84% male) completed the comprehensive psychosocial screener and questionnaires to gauge gender identity, traits, and sociocultural norm scores. A three-step latent profile analysis (LPA) was performed to identify latent profiles and their correlates.

**Results:**

LPA revealed six psychosocial risk profiles: (1) somewhat distressed overall (32%); (2) low distress (27%); (3) anger, hostility, and Type D (15%); (4) emotional distress and trauma (11%); (5) anxiety (9%); and (6) high overall distress (7%). Masculine traits and older age increased the odds to belong to the low distress profile (#2), while feminine traits and a feminine gender norm score increased the chance to belong to profiles with moderate to high distress. The effects of gender identity and feminine traits were sex dependent.

**Conclusion:**

The current study’s findings explain heterogeneity among patients with CHD by considering the joint occurrence of psychosocial risk factors, and the role of sex, age, and gender within those profiles. Being more sensitive to the roles that sex, gender, and an integrated set of risk factors play may ultimately improve treatment and adherence.

**Supplementary Information:**

The online version contains supplementary material available at 10.1007/s12529-023-10170-5.

## Introduction

A multitude of studies have highlighted the contribution of psychosocial distress to the risk of a worsened clinical prognosis and outcome of coronary heart disease (CHD) (e.g., [1–3]). Of note, this risk is independent of the well-known classical risk factors like smoking and hypertension, but of comparable size [4]. Furthermore, the distress experienced by cardiac patients is associated with a poorer health-related quality of life and well-being [5–7], and with more adverse events surrounding cardiac treatment [8]. The European Society of Cardiology (ESC) summarized these psychosocial risk factors in the guidelines for the prevention of cardiovascular disease [1], including depression, anxiety, Type D personality, chronic stress (i.e., perceived work and social stress/low social support), anger, traumatic stress, hostility, low socioeconomic status (SES), and psychiatric history [1].

Even though studies have established the single effects of these risk factors, they are often found to cluster [1, 9] which complicates risk assessment [10]. This within-person clustering may occur because of shared underlying mechanisms [11]. For example, the personality trait negative affectivity (NA) is thought to predispose individuals for the experience of depressive and anxiety symptoms [12, 13]. Further, a low SES is oftentimes characterized by higher stress levels [14–16], and seems to cluster with depression, social isolation, and hostility [17, 18]. Finally, shared underlying mechanisms, [e.g., 11] and disease pathways [19, 20] further hint at the necessity to study psychosocial risk factors on a within-person level. To date, the clustering of psychosocial risk factors mainly is studied at population-level, based on its co-occurrence. Very few studies have examined the within-person clustering (i.e., risk profiles). Taken together, more insight is needed into within-person psychosocial risk profiles, given that the joint effect may differ from the single risk factor effects [21]. Furthermore, studying individual differences within these psychosocial risk profiles may improve risk assessment and help develop personalized interventions.

Sex differences are important in explaining individual differences in the pathophysiological processes of cardiovascular disease [22, 23]. There are sex differences in the impact that the various classical risk factors, such as smoking, hypertension, and dyslipidemia, have. Biological analysis shows sex differences in plaque anatomy too, importantly linked to divergent risks for future cardiovascular events [24]. Moreover, epidemiological data show a poorer prognosis for female patients after myocardial infarction, as compared to men [25–27], although inconsistencies have been reported too [28, 29]. Women seem to become more susceptible to cardiovascular disease in the decades following menopause, which suggests a cardioprotective role for estrogen (blood pressure control through vasodilation, potentially altering vascular impact of systemic inflammation). Additionally, testosterone (low levels associated with increased systemic inflammation) may be involved as well [24].

Behavior-wise, sex differences are present in the context of cardiac rehabilitation (CR), with women being less likely to take part in CR programs [30]. Sex differences also exist in psychological risk factors, apparent in higher lifetime rates for depression [31] and generalized anxiety disorder (GAD) [32, 33], and increased susceptibility to the onset of (traumatic) stress-related psychiatric disorders in women [34], even though the number of experienced traumatic events may be higher in men [35]. Besides, men tend to have higher levels of hostile affect [36], which was also shown in an earlier latent profile analysis in patients with CHD [9].

Recently, both health research and care have drawn attention to the role of gender apart from sex differences. Like sex, gender is an important determining factor for health and well-being, as it affects healthcare-related behavior [37]. Gender is a multidimensional [38] sociocultural construct (Box 1), which is associated with an individual’s identity, behavioral tendencies, and sociocultural norms [39–41]. Gender identity entails how people perceive and display themselves concerning gender norms. Further, gender is thought to shape roles and behavior, albeit it more complex and prone to changes over time and place as compared to sex. In this context, gender is stereotypically distinguished by masculine (e.g., dominance, leadership) and feminine (e.g., sympathetic, gentle) traits. Sociocultural gender norms refer to attitudes and expectations that are produced by the society one is in, based on e.g. social interactions and cultural products. Previous research found that trait masculinity is associated with less overall distress, while feminine trait characteristics are related to higher levels of anxiety [42]. Importantly, gender differences also exist in physical health: for example, masculinity may be disadvantageous for men’s general health as their ideals may prevent them from using health services [43, 44]. Masculinity is also associated with an increased risk for CHD [45], while men that score higher on femininity may have a lower risk of CHD-related mortality [46]. A recent study found that in both men and women, masculine characteristics were related with an elevated risk to develop CHD, which was more pronounced in women [47]. Sex and gender have previously been found to interact, such that the effect of gender depends on one’s sex [39, 48], but more insight is needed to have a clearer understanding which role they both play in explaining individual differences in psychosocial risk factors among cardiac patients.

**Box 1:**
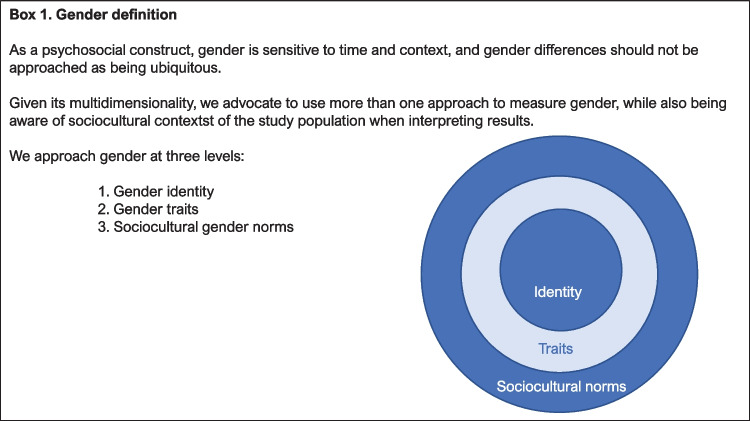
Gender definition

The current study thus has multiple aims: first, it investigated whether and how psychosocial risk factors cluster together in patients with CHD. Then, it was explored how sex and gender characterize these psychosocial risk profiles. Given that older age is associated with lower levels of distress [49, 50], the analyses will be adjusted for age. Based on previous research [34, 42], it was expected that feminine characteristics and female sex are associated with higher distress, while masculinity and male sex are expected to be related to lower levels of distress.

## Methods

### Procedure

The current Gender sub-study of the Tilburg Health Outcomes Registry of Emotional Stress after Coronary Intervention (THORESCI) is an add-on to the large, ongoing, longitudinal observational cohort study that started in December 2013. Patients who underwent either an acute (i.e., urgent) or elective (i.e., planned) percutaneous coronary intervention (PCI) for CHD are followed for 2 years [51, 52]. The Gender sub-study comprised an additional data collection (November 2020–January 2021), offered to original THORESCI participants (completed or still active in the main study). All participants either received an electronic invitation to fill out an electronic (~ 60%) or paper questionnaire (postal mail), together with an information letter and a form to provide informed consent, depending on their stated preference. Participation was voluntary. Eligibility criteria for the main study were mastering the Dutch language, both verbally and written, and the absence of any life-threatening comorbidities (e.g., metastasized cancer) or cognitive disorders. The study protocol and its amendments are in accordance with the Declaration of Helsinki and approved by the institutional medical ethical review board (METC Brabant, reference number NL46259.028.13). The current data analysis plan has previously been pre-registered on Open Science Framework (https://osf.io/z6tda/).

### Materials

#### Comprehensive Psychosocial Screening Instrument

The Comprehensive Psychosocial Screening Instrument [53], based on the ESC cardiovascular prevention guidelines, was administered at the same time as the gender-related questions. The survey instrument has 19 items that ask to report on eight pre-defined psychosocial risk factors that cover emotion, personality, and perceived stress domains, including depression (3 items), anxiety (2 items), perceived chronic stress (work stress (3 items), family stress (1 item)), trauma (1 item), Type D personality (negative affectivity [NA] and social inhibition [SI]; 4 items), anger (2 items), and hostility (3 items). All items are answered on a 4-point Likert scale (1 = ‘not at all’, 4 = ‘very much so’). The psychometric properties and validity of the instrument among two populations of patients with CHD were recently studied, which revealed that the screener had an adequate performance with fair to excellent levels of agreement with established full scales. The eight-factor structure of the instrument was also confirmed. Therefore, it was concluded that the screener is a quick and reliable tool to provide a good indication of the psychosocial risk status [53, 54].

#### Demographics and Medical Background Variables

##### Demographics

Demographic variables were obtained from self-report and the patients’ medical records, and included birth-assigned sex, age, educational level, and occupational status.

##### Clinical Characteristics

The clinical sample characteristics were obtained from the medical records at baseline, and included cardiac history (e.g., a previous PCI, coronary artery bypass grafting [CABG], or myocardial infarction [MI]), PCI indication (i.e., acute or elective), comorbidities (e.g., cancer, transient ischemic attack, kidney disease), and risk factors (e.g., hypertension, dyslipidemia). Lifestyle characteristics were obtained by self-report and included smoking (yes, no, previously) and physical activity (yes, no).

#### Gender

##### Subjective Gender Identity

A one-item prompt was included to assess to what extent a participant identified themselves as feminine, masculine, or neither of those. Participants could indicate this on a continuous scale that ranged from − 50 (100% masculine) via zero (neither) to 50 (100% feminine), which was recoded such that the scale ranged from 0 (masculine) to 100 (feminine), to ensure an easier interpretation.

##### Gender Traits

Given the more traditional sample (patients born on average mid-1950s), the condensed version of the Bem Sex Role Inventory (BSRI) [55] was used to examine gender traits. Characteristics that are typically viewed either as masculine (e.g., dominant; 6 items) or feminine (e.g., tender; 6 items) were endorsed on a 7-point Likert scale from *almost never true* to *almost always true*. In a previous study, the two-factor structure of the Dutch BSRI in confirmatory factor analysis was confirmed [56]. For both masculine and feminine traits, the total score was calculated by summing up the individual items. The internal consistency was good for both scales (masculine: *α* = 0.852 and feminine: *α* = 0.899). Continuous scores were analyzed for masculine and feminine traits separately.

##### Gender Norm Score

Several recommendations have been offered for a composite gender norm score capturing sociocultural gendered behaviors [41, 57, 58]. Based on this, and our own research [56], the current study combined civil status, occupational status, primary earnership, education level, and household task division to build a composite gender norm score [41, 57, 58]. The sum of the five gendered items was taken to compute the gender norm score, while allowing for two missing variables. In case of a missing value, the item was replaced by taking the mean of the gendered items that were available. The gender norm score concerns a continuous score which could range between 0 and 10, with a higher score indicating more female gender roles and a lower score more male gender roles. The pre-registration contains an explanation on the variables and the coding that were used (https://osf.io/z6tda/).

### Statistical Analysis

Patient characteristics were described for men and women separately and sex differences were assessed for categorical variables using chi-squared tests and for continuous variables using one-way ANOVAs. Point-biserial correlations and Pearson correlations were calculated to measure the associations between the gender and sex measures. We tested whether the gender norm score aligned with someone’s sex based on the sex-stratified median splits of the gender identity item and the gender norm score. These analyses were conducted in SPSS version 24 (IBM Corp., Armonk, NY, USA).

To answer the research questions, first, a latent profile analysis (LPA) was performed in LatentGOLD v6.0 [59]. LPA allows for the exploration of the most optimal within-person grouping of risk factors to identify latent profiles [60]. The eight psychosocial risk factors were entered and the fit of 10 subsequent models with an increasing number of classes was tested (1–10). Information criteria (i.e., the Akaike information criterion [AIC], AIC3, and the Bayesian information criteria [BIC]) and the Vuong-Lo-Mendell-Rubin (VLMR) test were used to determine the best possible model [61]. Lower AIC and BIC values of a specific model indicate a better fit [62]. Previous research concluded that AIC3 is a better criterion as compared to the other criteria [63, 64]. The VLMR test comes with a significance level, indicating whether the larger, more detailed, model was an improvement of fit to the data when compared to models with a one class difference (i.e., a 2-class model gets compared to a 1-class model). Probability scores of each possible class membership (ranging from 0.00 to 1.00) were assigned to every individual by considering the classification inaccuracy as provided by the LPA. Participants were automatically assigned a profile based on their highest probability score.

Then, the three-step LPA was used to examine correlates of the profiles, as recommended [65]. Correlates of the resulting profiles were examined in the third and final step, in a multinomial logit model that also considered classification errors. Then, associations of sex and gender variables and their interaction were hierarchically tested followed by the inclusion of age as a covariate. While the overall Wald statistic informs about predictor differences between all classes, individual profile odds ratios are indicative of a specific association. All analyses were done based on a 5% significance level (*p* < 0.05). An in-depth explanation on the analysis can be found in our pre-registration plan (https://osf.io/z6tda/).

## Results

### Sample Characteristics

In total, 1332 THORESCI participants were invited to take part in the Gender sub-study survey. The 532 participants who completed the survey had a mean age of 68.18 (*SD* = 8.93), 16% was female (Table 1), and 65% had received an acute index PCI (Table S1). When comparing the current sample to the main cohort with regard to cardiac history (*χ*^2^ = 0.34) and comorbidity (*χ*^2^ = 0.67), no significant differences were found (*p* > 0.05). Other medical background and lifestyle characteristics were stratified by sex which likewise revealed no significant differences between men and women (Table S1).

**Table 1:** Sex-stratified demographic, psychosocial, and gender characteristics

		Women		Men			
	*N*	%/mean	*N*/SD	%/mean	*N*/SD	Test-value	*p* value
***Demographics***							
* Sex*	532	16%	86	84%	446		
* Age*	532	67.23	9.21	68.37	8.87	0.196	0.658
***Psychosocial risk factors***							
Depression (3 items)	493	5.42	1.60	4.74	1.81	9.67	**0.002**
Anxiety (2 items)	489	2.79	0.93	2.79	1.09	0.001	0.850
Negative affectivity (2 items)	496	2.86	1.10	2.78	1.08	0.457	0.603
Social inhibition (2 items)	492	2.88	1.19	3.00	1.27	0.372	0.436
Work stress (3 items)	443	4.16	1.34	4.16	1.53	0.094	0.840
Family stress (1 item)	487	1.36	0.73	1.28	0.60	1.24	0.333
Anger (2 items)	490	3.04	1.02	3.04	1.12	0.904	0.219
Hostility (3 items)	491	4.40	1.37	4.63	1.55	0.055	0.978
Trauma (1 item)	489	1.38	0.65	1.31	0.66	1.62	0.402
***Gendered items***							
** Gender traits**							
Masculinity	495	26.5	7.04	29.5	6.47	13.2	**0.001**
Femininity	496	33.2	6.92	31.7	6.13	3.99	**0.046**
** Gender identity**							
Gender identification	433	87.6	14.9	14.1	19.2	875	**< 0.001**
** Gender norm score**	433	4.97	1.38	3.00	1.68	78.0	**< 0.001**
* Occupational status*	429					13.0	**0.001**
Full-time (0)		6%	4	23%	85		
Jobless/retired (1)		70%	44	65%	237		
Part-time (2)		24%	15	12%	44		
* Primary earner status*	410					61.6	**< 0.001**
Primary earner (0)		34%	20	67%	236		
Equal earners (1)		22%	13	26%	91		
Not primary earner (2)		43%	25	7%	25		
* Educational level*^*a*^	503					1.48	0.476
Middle voc. training or + (0)		40%	31	45%	192		
College or similar (1)		30%	23	31%	134		
High school or − (2)		30%	23	23	100		
* Household division*	393					43.4	**< 0.001**
Partner/other does most (0)		10%	5	47%	162		
Shared (1)		27	14	31%	107		
I do most (> 60%) (2)		63%	32	21%	73		
* Civil status*	529					9.88	**0.007**
With partner (0)		72%	61	86%	380		
Single (1)		9%	8	5%	20		
Divorced/widowed (2)		19%	16	10%	44		

^a^Based on baseline values; Bold, *p* < 0.05

### Psychosocial Risk Factors

Sex-stratified means and standard deviations of each psychosocial screening scale separately are displayed in Table 1. The total scores of the nine screening scales (eight risk factors) were used in the first step of the LPA. Sex differences were detected for the depression subscale only, with women scoring significantly higher than men (*F*(1, 491) = 9.28, *p* = 0.002).

### Gender and Sex

**Fig. 1:**
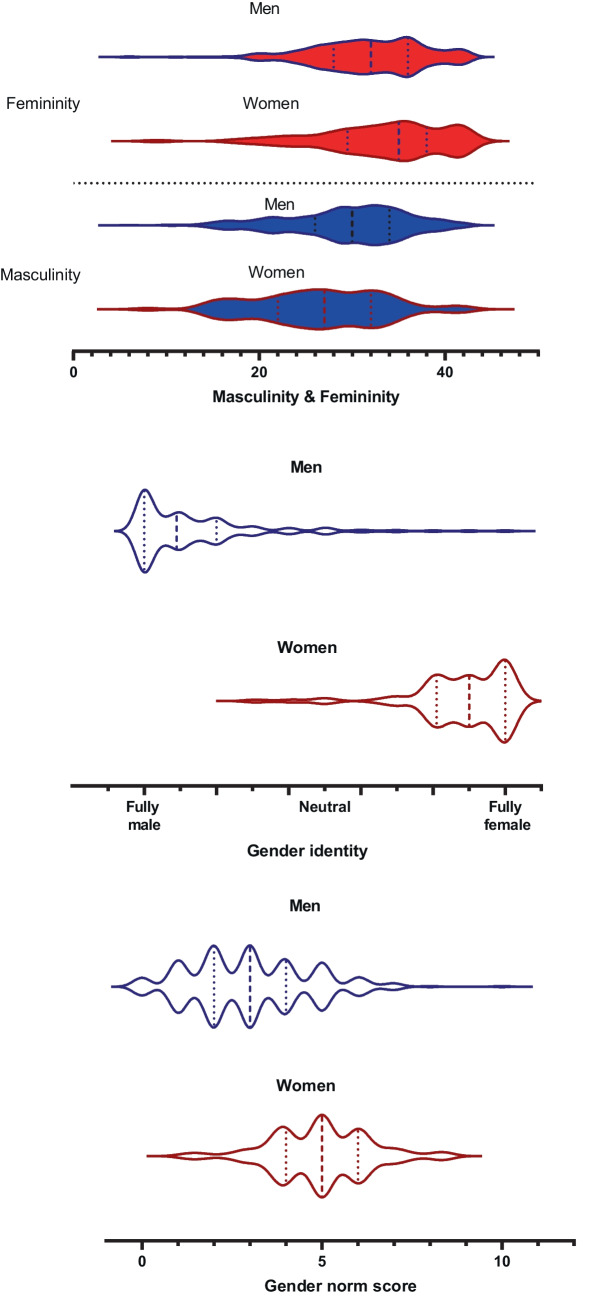
Sex-stratified violin plots showing the data distribution for gender traits (i.e., masculine and feminine traits), gender identity, and gender norm score. Striped line, male/female mean. Dotted lines, ± 1SD from the respective means

Sex-stratified descriptive statistics of each gender measure are presented in Table 1 and Fig. 1. All gender measures showed significant sex differences (*p* < 0.05), with women scoring higher on feminine-related identity, traits, and sociocultural norm score, and men scoring higher on masculinity. In addition, alignment with sex was incomplete, with 52–57% of men showing no alignment of gender with sex, and 32–39% of women showing this (Fig. 1).

Correlational analysis on sex and each gender measure revealed a strong, congruent relationship between sex and gender identity (*r* = 0.812, *p* < 0.01), and a moderate congruent relationship between sex and gender norm score (*r* = 0.391, *p* < 0.01). Further, moderate positive relationships were found between the gender traits masculinity and femininity as assessed with the BSRI (*r* = 0.393, *p* < 0.01) and between the gender norm score and gender identity (*r* = 0.359, *p* < 0.01). Other relationships were either nonsignificant, weak, or both. All correlations are displayed in Table S2.

### Latent Profile Analysis

Model fit statistics of the LPA are displayed in Table S3. The three fit statistics pointed at different models. While the VLMR test indicated a 5-profile model was sufficient to describe the data well, the AIC3 suggested a 6-profile model, and the BIC suggested a 4-class model. Content analysis [66] revealed the 4-profile model lacked detail, as it merely discriminated between low, moderate (two profiles), and high overall levels of distress. The 5-profile and 6-profile model had some similarities, with the 6-profile model containing more detail as it revealed a profile characterized by high anxiety and high perceived work stress, which is a clinically relevant profile. Based on these characteristics, as well as the lower AIC3 [63, 64], the analysis was continued with a 6-profile model.

**Fig. 2:**
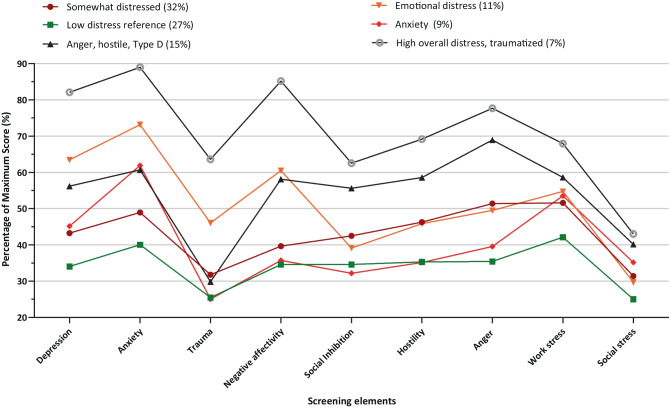
Latent class profiles

The profiles are displayed in Fig. 2. It is relevant to note that the overall prevalence of women is 16% in this cohort. The first and largest profile (32%; 16% female) was characterized by some distress, followed by the second profile which contained 27% of participants (12% female) and was defined by low levels of distress. The third profile consisted of individuals that mainly scored high on anxiety, hostility, and Type D personality (15%; 19% female). The fourth profile (11%) contained most women (25%) as compared to the other profiles and was characterized by emotional distress (i.e., depression, anxiety, negative affectivity) and trauma. The fifth profile (9%; 16% female) was known for anxiety only. The smallest and sixth profile (7%; 16% female) was characterized by high overall distress, expressed in emotional problems and trauma. It was found that perceived work stress (*R*^2^ = 15%) and social stress (*R*^2^ = 14%) were least contributing, while negative affectivity (*R*^2^ = 66%), depression (*R*^2^ = 56%), and anger (*R*^2^ = 54%) were contributing most.

### Latent Screening Profiles, Sex, and Gender

**Fig. 3:**
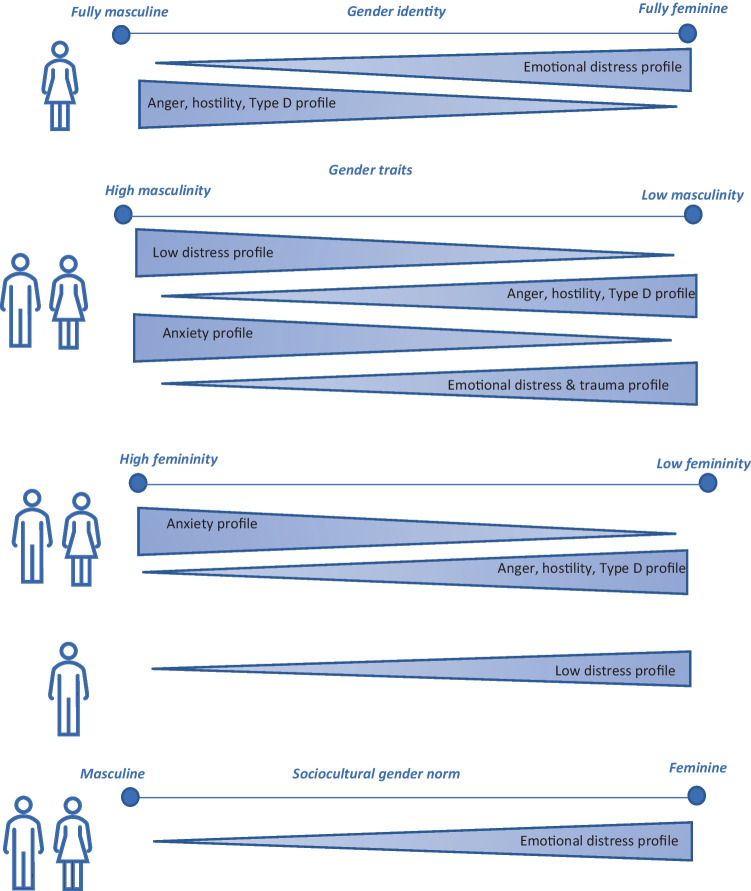
Summary of the profile memberships for each gender measure. Symbols on the left indicate main effects (both male and female) or interaction effects (male or female)

The sex and gender measures were then associated with the different latent screening profiles. The odds of belonging to either of the latent profiles for each of the gender measures and the demographics (i.e., sex and age) are displayed in Table 2. Table S4 contains the unadjusted main effects of each gender measure. Furthermore, in Fig. 3, the main and interaction effects for each gender measure are summarized.

**Table 2:** Adjusted associations (in odds) with class membership for sex, gender, and the interaction between sex and gender

	(1) Somewhat distressed (*n* = 170)	(2) Low distress—reference (*n* = 142)	(3) Anger, hostility, Type D personality (*n* = 81)	(4) Emotional distress (*n* = 57)	(5) Anxiety (*n* = 45)	(6) High overall distress, traumatized (*n* = 37)	Overall statistics	
	Odds						Wald	*p* value
***Demographics***								
Sex (women)	0.68 (0.34–1.35)	0.57 (0.32–1.02)	1.15 (0.60–2.19)	**2.04 (1.06–3.92)**	1.52 (0.74–3.13)	0.73 (0.30–1.78)	9.978	0.076
Age	1.03 (0.999–1.07)	**1.05 (1.02–1.07)**	**0.96 (0.93–0.99)**	**1.05 (1.00–1.11)**	0.96 (0.92–1.00)	0.96 (0.92–1.00)	28.490	< *0.001*
***Gender identity***^***a***^								
Gender identity	1.01 (0.999–1.02)	0.997 (0.98–1.01)	0.998 (0.986–1.01)	1.01 (0.99–1.03)	0.999 (0.98–1.01)	0.990 (0.97–1.01)	2.937	0.710
Sex (women)	1.42 (0.88–2.26)	1.36 (0.79–2.36)	0.67 (0.42–1.07)	1.30 (0.49–3.44)	0.75 (0.37–1.54)	0.79 (0.43–1.46)	6.816	0.230
Gender identity*sex	0.999 (0.988–1.01)	0.990 (0.97–1.01)	**1.02 (1.003–1.03)**	**0.98 (0.96–0.999)**	1.004 (0.989–1.02)	0.99 (0.97–1.007)	9.811	0.081
***Gender norms***^***a***^								
Gender norm score	1.36 (0.88–2.10)	1.04 (0.75–1.43)	0.66 (0.42–1.02)	1.80 (0.97–3.35)	0.74 (0.37–1.51)	0.80 (0.60–1.14)	6.872	0.230
Sex (women)	1.63 (0.93–2.86)	1.30 (0.86–1.97)	0.61 (0.34–1.08)	1.56 (0.40–5.99)	0.59 (0.31–1.12)	0.84 (0.44–1.62)	6.461	0.260
Gender norm score*sex	0.68 (0.45–1.05)	0.84 (0.61–1.15)	1.56 (0.97–2.50)	0.64 (0.32–1.29)	1.29 (0.64–2.62)	0.73 (0.51–1.05)	7.107	0.210
***Masc. gender traits***^***a***^								
Masculinity	1.06 (0.97–1.15)	1.08 (0.997–1.17)	0.90 (0.81–1.003)	0.99 (0.93–1.05)	1.05 (0.98–1.12)	**0.94 (0.89–0.98)**	13.576	*0.019*
Sex (women)	1.06 (0.72–1.58)	1.17 (0.83–1.63)	1.34 (0.64–2.80)	0.71 (0.43–1.18)	0.75 (0.51–1.12)	1.12 (0.62–2.03)	5.455	0.360
Masculinity*sex	0.97 (0.89–1.05)	0.98 (0.90–1.06)	1.06 (0.96–1.18)	1.02 (0.96–1.08)	1.02 (0.96–1.09)	1.04 (0.988–1.09)	6.991	0.220
***Fem. gender traits***^***a***^								
Femininity	**0.85 (0.75–0.97)**	**1.17 (1.08–1.27)**	**0.86 (0.79–0.94)**	1.14 (0.97–1.35)	**1.12 (1.00–1.25)**	0.92 (0.84–1.01)	23.328	< *0.001*
Sex (women)	1.69 (0.83–3.44)	**1.64 (1.21–2.22)**	0.67 (0.42–1.06)	0.90 (0.43–1.89)	0.67 (0.40–1.12)	0.90 (0.54–1.50)	17.717	*0.003*
Femininity*sex	1.14 (0.999–1.30)	**0.86 (0.79–0.93)**	1.07 (0.98–1.17)	0.89 (0.75–1.05)	0.98 (0.88–1.11)	0.92 (0.84–1.01)	5.839	*0.007*

^a^All gender models are adjusted for age; estimates and CI values close to 1.00 have been displayed with an additional decimal, for easy interpretation. **Bold faced**, significant effect on individual profile; *italic*, significant overall result, trait is affecting the majority of profiles (*p* < 0.05)

#### Demographics: Sex and Age

Sex as a predictor of latent profile membership was tested first. Analyses revealed that only one profile was characterized by a relative lack of men; i.e., men had a lower likelihood to belong to the fourth profile (i.e., emotional distress).

Age significantly predicted class membership for all classes (all *p* < 0.01), with older patients having a higher probability of belonging to profiles 2 and 4. This is interesting, as these are the low distress, and the emotional distress profiles. Younger patients were more likely to belong to the third profile (i.e., anger, hostility, and Type D personality). The associations of age with the odds to belong to the emotional stress (#4; OR = 1.15; 95% CI: 1.02–1.31) or high overall distress profiles (#6; 1.09; 95% CI: 1.004–1.17) were more pronounced in women. These effects largely remained the same (data not shown) in the gender models.

#### Subjective Gender Identity

There were no significant main effects of gender identity on the psychosocial risk profiles. When examining whether the effect of gender identity was different for men vs. women, there were two significant interactions. While in women, a higher gender identity score (i.e., the more feminine) lowered the probability to belong to profile 3 (anger, hostility, Type D), in men, such relationship was absent. The second interaction pertained to the emotional stress profile (#4), with women with a higher feminine identity being more likely to belong to profile 4, while in men this relationship was absent.

#### Gender Norm Score

In the unadjusted analysis, a higher (i.e., more feminine) gender norm score significantly increased the odds to belong to profile 4, characterized by emotional stress (OR = 1.38; 95% CI: 1.12–1.71; Wald = 12.697; *p* = 0.026; Table S4). When considering the effects of sex, age, and the interaction between sex and the gender norm score, this effect disappeared. Nevertheless, the association with profile 4 remained relatively large and approached significance. Because of the explorative nature of the current study, this adjusted effect remains of potential interest. The effect of the gender norm score did not depend on one’s sex.

#### Gender Traits

The unadjusted main effects model (Table S4) revealed that masculine traits were significantly related to risk profile membership (Wald = 22.748; *p* < 0.001), with an increased odds to belong to the low distress profile (#2; OR = 1.06; 95% CI: 1.02–1.10) and the anxiety profile (#5; OR = 1.06; 95% CI: 1.004–1.12), and lowered odds to belong to profile 3 (i.e., anger, hostility, and Type D personality; OR = 0.95; 95% CI: 0.91–0.99) and profile 6 (i.e., high overall distress; OR = 0.93; 95% CI: 0.89–0.98). In the full models, including sex, age, and the interaction between masculinity and sex, only the association with profile 6 remained significant. The effect of masculinity did not depend on sex (Wald = 6.991, *p* = 0.220).

Unadjusted main effects analysis for feminine traits showed associations with profile membership as well. A higher level of femininity was associated with reduced likelihood of belonging to profile 3 (‘anger, hostility, Type D’; OR = 0.93; 95% CI: 0.89–0.97), while increasing the odds to belong to the profile characterized by elevated levels of anxiety (#5; OR = 1.10; 95% CI: 1.03–1.16). In the full models, odds remained similar, but significance shifted a bit: the association with profile #5 reduced to trend level and the addition of a significant association for a smaller likelihood to belong to profile #1 (‘moderate stress’). The effect of femininity differed between men and women (Wald = 15.839, *p* < 0.01) with men scoring higher on femininity having a reduced likelihood to belong to the low distress profile (#2).

## Discussion

The current study explored the within-person clustering of psychosocial risk factors among patients with CHD and examined how sex and gender characterized these profiles, while adjusting for patients’ age. LPA resulted in six distinct psychosocial risk profiles characterized by individual differences in personality and emotional distress. With respect to sex and gender differences, several conclusions were reached. Men were less likely to belong to the emotional distress profile (#4). For gender identity, it was found that in women only, a more feminine score was associated with the ‘emotional distress’ profile, and a more masculine score was associated to membership to the ‘anger, hostility, Type D’ profile, which is typically viewed as the psychological male CHD patient archetype. At the trait level, higher scores on trait masculinity were associated with the ‘low distress reference’ profile, and the ‘anxiety’ profile, while low trait masculinity was associated with the ‘anger, hostility, Type D’ profile and the ‘overall distress and traumatized’ profile; all independent of sex. The femininity trait was positively associated with the ‘anxiety’ profile, and lower femininity was associated with the ‘anger, hostility, Type D’ profile. In men, increased femininity related to a lower likelihood of belonging to the low distress profile. In terms of sociocultural norms, femininity was associated with the ‘emotional distress’ profile, which was similar in men and women (see Fig. 3 for a visual summary).

In line with recommendations from prior studies [22, 57, 58], gender was operationalized at multiple levels to reflect its expression dynamics. Most previous research, though, used the traits questionnaire only. Our findings are in accordance with extant research reporting masculine gender to be related to more robust psychological functioning [42], and femininity to increase the risk of emotional distress slightly [67]. Importantly, previous research has emphasized the importance of attending to the intersecting roles of gender and sex [68], also in cardiology [69]. When examining the interaction of sex by gender, results showed effects that were different for men and women, and different for various gender facets; i.e., while more feminine men (trait) were less likely to belong to the low distress profile (#2), a more feminine gender identity amplified the general female tendency to experience more emotional distress, but only in women. It is important to demonstrate these intersectional differences exist for non-traditional risk factors, such as psychosocial factors. A previous landmark study showed that patients with CHD who were more feminine in their personality and social roles had an increased risk of a recurrent event, while event rates in men and women were equal. Their findings also revealed that anxiety was the only variable that could explain this increased risk [22]. Note that only the separate effects of masculine and feminine traits were examined, while they can also co-occur, or both be absent. Interestingly, low femininity also has been associated with an increased risk of cardiac mortality [46], highlighting the need for additional research on the potential U-shaped risk observed for femininity.

Interestingly, median sociocultural gender norm scores were low (3; men) to moderate (5; women), suggesting that the women in the current sample were more defined by masculine-gendered characteristics. For instance, a substantial proportion of women indicated to have primary earner status, a higher education, and a partner, contributing to a lower index score. Other attributes may have influenced these findings though; for instance, poorer health status also could lead to spending less time on household tasks [47] which would be coded as masculine. Moreover, in older age, masculinity and femininity differences may diminish, possibly owing to declines in sex hormones.

Previous research summarized by Connelly et al. (67) showed the importance of gender, both directly, as a predictor CHD, and indirectly through various risk factors. The authors suggest that gendered domains, including psychological factors, may mediate the pathways between gender identity and cardiac risk [69]. Moreover, patient-reported health outcomes among female cardiac patients following myocardial infarction were found to be better predicted by psychosocial factors than by classical cardiac risk factors [70], as well as gendered characteristics such as femininity and household tasks [71]. These women may lack sufficient psychosocial resources while also being burdened with gender-related tasks [72]. This advocates for increased attention to both psychosocial risk and gender in cardiac practice and research since their influence may be more pronounced than initially assumed. In the context of the current study, these relationships between gender and CHD risk may be even more augmented when considering psychosocial risk profiles rather than the usually studied individual risk factor effects.

The current study highlighting the importance to consider gender and its intersection with sex and the effects on psychosocial risk profiles within the context of cardiology leaves a promising avenue for future directions. Future research could further examine how psychosocial risk profiles underlie the mechanism between gender and CHD, as well as how these mechanisms differ for men and women. Furthermore, it is likewise encouraged to study how the clustering of psychosocial risk factors in risk profiles relates to other cardiac risk factors in terms of size.

The current results also give rise to several clinical implications. With the rise of examining sex differences in cardiovascular risk and outcome prediction, the current results show that it may be worthwhile to include an assessment of gender as well, as gender is not covered by the effects of sex, and the effects of gender may also differ depending on whether someone is male or female. Interventions within the context of cardiac rehabilitation (CR) thus should not only focus on treating these joint psychological risk factors, but also be more sensitive to gender and sex differences. Exploring gender-related experiences during the therapeutic process could aid psychological growth, as is done in Gender Aware Therapy (GAT), which includes gender as an integral aspect of the treatment, uses a client’s social context to gain an insight into their burden [73]. It was recommended to assess both topic and dynamic of gender during the therapeutic process and advocate an intersectional approach [74].

Sex differences appear in participation in, and completion of, CR programs, with women adhering less to the recommendation to take part in CR [30]. Moreover, femininity traits have been related to poorer physical functioning (6-min walk test) [75], stressing the need for more knowledge on the roles of sex and femininity in adherence to, and capacity in, CR programs. Likewise, the role of masculinity should receive more attention, too. It was previously suggested that during CR men may be focused on reclaiming their masculine identity after being affected by heart disease [76–78]. A previous study on health-promoting behavior highlighted the importance of taking gendered backgrounds of health behavior into consideration, e.g., by challenging masculine beliefs and the reappraisal of specific situations and activities that are threatening to masculinity [79]. Future research could provide more insight by examining whether a sex- and gender-sensitive CR approach would be beneficial in terms of physical and psychological outcomes.

The current study was not without limitations. Firstly, a gender norm score that was derived from research in working age populations [42, 57, 58] was used, which may have been less than ideal for the current, older-aged sample, which was for the majority retired, and more often widowed. The meaning of retirement has received little attention in health research [80]. Retirement was placed in the middle category as a less masculine societal gender norm. While moving in the right direction in terms of hypothesized emotional distress, this may also have been too simplistic, as masculine men seem conflicted and sometimes depressed by retirement [76, 81]. The most convincing effects were found for gender traits as measured by the BSRI [55]. Though widely used, it has also been criticized for its stereotypical, and arguably outdated, classification [82], among others because it was developed in a predominantly white population during the mid-twentieth century [46, 83]. With our sample consisting of participants born in the mid-twentieth century (on average 1954), and the vast majority belonging to a white, heterosexual population, they likely fit the traditional Western gender roles as measured with the BSRI. Nevertheless, the importance of gender being subject to change needs to be emphasized, as it can vary between generations, contexts, and cultures [39], and across the life course [84]. Additionally, a previous meta-analysis found that heterosexual men and women conform more to self-attributed gender roles [85]. Even though a strong positive association between sex and gender identity was found, for many participants their gender identity did not align fully with their sex. It should be noted that the current study only enabled participants to express their self-defined gender identification by either masculine, feminine, or in-between (i.e., undifferentiated), not allowing for other options, like gender fluidity [86]. Further, gender identity may vary over time and contexts [39, 87]. One item on gender identification may thus insufficiently capture the full scope of gender identity. Another limitation concerns the generalizability of the current results. The current study concerns a smaller subsample of a larger cohort study which in theory may lead to selection bias. However, after comparing the current sample to the sample of the main study, it was concluded that there were no differences in cardiac history and comorbid diseases between the two samples. This led us to believe that the selection bias is limited. Gauging psychosocial risk is important for all patient groups with cardiovascular disease. As the current paper was based solely on patients with CHD, future research would benefit from including other patient groups (e.g., atrial fibrillation, heart failure, or (M)INOCA), as their psychosocial risk status and experiences may differ as a function of impact of diagnosis, disease characteristics, or stage. Furthermore, the lack of diversity in the current sample (majority white, middle-aged men), though characteristic for the PCI patient population [88], may limit the generalizability to other cardiac patient samples that contain more women, or more people from other ethnic backgrounds. The small proportion of women did not come as a surprise, given that women less often undergo revascularization for acute coronary syndrome [89, 90] and are generally underrepresented in research that includes PCI patients [88]. Furthermore, minority groups tend to be represented less in the current survey study, which may be related to language barriers. Lastly, there are multiple variables associated with psychosocial risk which could have controlled for. The 2016 ESC prevention guidelines [1] were followed which limited the psychosocial variables to those listed in the guidelines. Additionally, other important variables such as hormonal status (e.g., menopause) were missing in the current study. Nonetheless, the majority (77%) of women were aged over 60. With the average onset of menopause in Western countries lying around age 50 [91], and over 90% of women experiencing cessation of menses by the age of 55 [92], it is fairly safe to speculate that menopausal effects in our study were limited. Finally, several medical background and lifestyle covariates were previously added which led to inflated confidence intervals and unreliable estimates. These issues were described in our pre-registration. When examining sex differences in medical background and lifestyle variables, results revealed there were no significant differences between men and women. For that reason, only age was entered as a covariate given its proven relationship with psychosocial distress [49, 50] but it is important to acknowledge that future research should include enough participants to reach sufficient power to include more covariates.

As for strengths, our within-person approach to psychological risk and the multifaceted approach to gender were novel. Moreover, the current study proves the importance of looking beyond sex differences as gender seemed to characterize profile membership more noticeably, which is concurrent with previous findings in patients with heart disease [22]. Instead of taking a single measurement approach to gender, the role of multiple aspects of gender and their influence on psychosocial risk was explored, which could lead to more refinement in screening and treatment options. Our findings contribute to an emerging understanding of how gender characteristics intersect with other determinants of mental and physical health.

In conclusion, six distinct psychological risk profiles were revealed based on eight established psychological risk factors [1]. Overall, older age and masculinity increased the odds of belonging to the lower distress profiles. Male sex lowered the odds of class membership to the profile characterized by emotional distress. The main effects of masculine traits, feminine traits, and the gender norm score were found to influence different risk profiles, of which findings were generally in accordance with previous research. Importantly, sex-dependent effects of gender identity and feminine traits were found. The results of the current study explain part of the heterogeneity in risk prediction among patients with CHD: considering the joint occurrence of psychosocial risk factors, as well as sex and gender differences within psychosocial risk profiles could ultimately improve treatment and CR enrollment by being more sensitive to the roles that sex and gender play.

### Supplementary Information

Below is the link to the electronic supplementary material.

Supplementary file1 (DOCX 38 KB)

## Data Availability

The metadata are accessible via https://dataverse.nl/dataset.xhtml?persistentId=doi:10.34894/TBNLAW. Data are available upon reasonable request.
