# Supplementary material for: Sex and Gender Differences in Psychosocial Risk Profiles Among Patients with Coronary Heart Disease — the THORESCI-Gender Study
Source: Int J Behav Med. 2023 May 11;31(1):130–44. doi: 10.1007/s12529-023-10170-5 (PMC10803502; doi:10.1007/s12529-023-10170-5)
Supplement: Supplementary file 1 — Supplementary file1 (DOCX 38 KB) [file 12529_2023_10170_MOESM1_ESM.docx]

**Sex and gender differences in psychosocial risk profiles among heart disease patients**

**Supplemental results**

*Descriptive statistics*

***Gender and sex*** - With regards to the separate gendered items that were used to establish the gender norm score, only the gendered item that did not differ between women and men (*p* = .476) was educational level. For the other four items, the more socio-culturally viewed feminine options were more often assigned to women whereas the socio-culturally viewed masculine options were more often accredited to men (*p* < .01; see Table 1) Additionally, analysis on alignment with the gender norm score for both sexes revealed that 57% of male participants’ gender norm score did not align with their sex (median split of 3), whereas for female participants this was 34% (median split of 5; see Figure S1). The median splits for gender identity were 9 and 90 for men and women respectively, which revealed that the gender identity of 52% of men and 39% of women did not align with their indicated sex (Figure S1). According to expectations, significant differences in gender identity were found between men and women (*M*_female_ = 87.64, *SD* = 14.87; *M*_male_ =14.07, SD = 19.24; *F*(1, 454) = 875.497, *p* < .001; Table 1).

**Supplemental table S1**. Sex stratified clinical and lifestyle characteristics.

|  |  | Women | | Men | | Test- | p-value |
| --- | --- | --- | --- | --- | --- | --- | --- |
|  | N | %/Mean | N/SD | %/Mean | N/SD | value |  |
| ***Clinical Characteristics^a^*** |  |  |  |  |  |  |  |
| PCI indication (% acute) | 528 | 64% | 54 | 65% | 289 | .091 | .762 |
| Cardiac history^1^ | 532 | 35% | 30 | 44% | 196 | 2.42 | .120 |
| Comorbid diseases^2^ | 532 | 49& | 42 | 38% | 168 | 3.76 | .052 |
| Hypertension | 532 | 51% | 44 | 41% | 181 | 3.31 | .069 |
| Hypercholesterolemia | 532 | 39% | 33 | 34% | 151 | .650 | .420 |
| Diabetes | 532 | 20% | 17 | 17% | 76 | .372 | .542 |
| ***Lifestyle Characteristics^a^*** |  |  |  |  |  |  |  |
| Smoker [yes] | 439 | 9% | 8 | 9% | 39 | .046 | .830 |
| Physically active [yes] | 520 | 69% | 58 | 70% | 307 | .063 | .802 |

^a^Based on the baseline values

^1^Previous myocardial infarction, coronary artery bypass grafting, percutaneous coronary intervention, pacemaker, heart failure, atrial fibrillation ^2^Diabetes, COPD, anemia, liver disease, kidney disease, cancer in the past 5 years, rheumatoid arthritis

**Supplemental table S2**. Point-biserial (sex – gender) and Pearson correlations (gender – gender).

|  | Sex | Gender identity | | Gender norm score | | Masculinity | | Femininity | |
| --- | --- | --- | --- | --- | --- | --- | --- | --- | --- |
| Sex | 1.000 | |  | |  | |  | |  |
| Gender Identity | .812** | | 1.000 | |  | |  | |  |
| Gender norm score | .391** | | .359** | | 1.000 | |  | |  |
| Masculinity | -.162* | | -.150* | | -.153** | | 1.000 | |  |
| Femininity | .090* | | .055 | | .030 | | .393** | | 1.000 |

Note: ** correlation is significant at the .01 level; * correlation is significant at the .05 level

**Supplemental Table S3**. Latent profile analysis model fit comparison

|  | Statistics | | | | | | | | |  |
| --- | --- | --- | --- | --- | --- | --- | --- | --- | --- | --- |
| Model | LL | BIC (LL) | AIC3 (LL) | Npar | df | VLMR | *p* | Class. Err. | Entropy R^2^ | Bootstrapped p-values |
| 1-profile | -5554.8333 | 11429.7755 | 11262.6667 | 51 | 481 |  |  | .000 | 1.000 | 5,2e-944 |
| 2-profile | -5168.0543 | 10718.9893 | 10519.1087 | 61 | 471 | 783.9485 | < .01 | .076 | .759 | 1,6e-795 |
| 3-profile | -5072.6637 | 10590.9691 | 10358.3274 | 71 | 461 | 193.7311 | < .01 | .116 | .734 | 4,7e-763 |
| 4-profile | -5036.3985 | *10581.2052* | 10315.7971 | 81 | 451 | 74.3781 | < .01 | .176 | .680 | 6,0e-754 |
| 5-profile | -5015.5394 | 10602.2534 | 10304.0788 | 91 | 441 | *42.6942* | .020 | .203 | .665 | **6,1e-751** |
| **6-profile** | **-4997.4124** | **10628.7658** | ***10297.8248*** | **101** | **431** | **36.2619** | **.080** | **.234** | **.653** | 4,9e-749 |
| 7-profile | -4984.1027 | 10664.9129 | 10301.2054 | 111 | 421 | 33.4089 | .04 | .248 | .658 | 4,6e-749 |

The chosen model is presented in bold. We evaluated its fit based on the *BIC*, *AIC3, VLMR* and content. LL = log likelihood; Npar = number of estimated parameters, VLMR = Vuong-Lo-Mendell-Rubin test

**Supplemental Table S4**. Associations (in odds) with class membership for the separate unadjusted main effects of gender

|  | Profile 1  (n = 170) | Profile 2  (n = 142) | Profile 3  (n = 81) | Profile 4  (n = 57) | Profile 5  (n = 45) | Profile 6  (n = 37) | Overall statistics | |
| --- | --- | --- | --- | --- | --- | --- | --- | --- |
| ***Gender*** | Odds | | | | | | Wald | p-value |
| Gender identity | 1.00 (0.993 – 1.007) | 0.991 (0.98 – 1.001) | 1.003 (0.996 – 1.01) | 1.008 (0.999 – 1.02) | 1.004 (0.994 – 1.01) | 0.995 (0.98 – 1.006) | 6.238 | .280 |
| Gender norm score | 0.96 (0.80 – 1.14) | 0.88 (0.78 – 1.00) | 0.90 (0.75 – 1.07) | **1.38 (1.12 – 1.71)** | 0.95 (0.75 – 1.22) | 0.997 (0.83 – 1.20) | 12.697 | *.026* |
| Masculine traits | 1.03 (0.991 – 1.07) | **1.06 (1.02 – 1.10)** | **0.95 (0.91 - 0.991)** | 0.98 (0.93 – 1.03) | **1.06 (1.004 – 1.12)** | **0.93 (0.89 - 0.98)** | 22.748 | *< .001* |
| Feminine traits | 0.96 (0.92 – 1.005) | 1.02 (0.97 – 1.06) | **0.93 (0.89 - 0.97)** | 1.02 (0.97 – 1.08) | **1.10 (1.03 – 1.16)** | 0.98 (0.94 – 1.03) | 17.214 | *.004* |

Note: ***^a^*** Estimates and CI values close to 1.00 have been displayed with an additional decimal, for easy interpretation. **Bold faced** = significant effect on individual profile; *italic* = significant overall result, trait is affecting the majority of profiles (*p* < .05)
